# Serum and urinary concentrations of arsenic, beryllium, cadmium and lead after an aerobic training period of six months in aerobic athletes and sedentary people

**DOI:** 10.1186/s12970-020-00372-7

**Published:** 2020-08-17

**Authors:** Diego Muñoz, Francisco J. Grijota, Ignacio Bartolomé, Jesús Siquier-Coll, Víctor Toro-Román, Marcos Maynar

**Affiliations:** 1 Sport Sciences Faculty, University of Extremadura, Avenida de la Universidad s/n, 10003 Cáceres, Spain; 2 Education Faculty, University of Extremadura, Avenida de la Universidad s/n, 10003 Cáceres, Spain

**Keywords:** Toxic metals, Exercise, Excretion, Blood

## Abstract

**Aim:**

The aim of the present study was to evaluate the possible effect of a period of 6 months of aerobic physical training on serum and urinary concentrations of arsenic (As), beryllium (Be), cadmium (Cd) and lead (Pb), potentially toxic minerals.

**Methods:**

Twenty-four well-trained, long distance runners (AG), were recruited at the start of their training period. They had been performing training regularly for the previous 2 years, recording an average volume of 120 km per week of rigorous aerobic exercise aimed at high-level competitions (1500 and 5000 m race modalities). Twenty-six untrained, sedentary participants constituted the control group (CG). All participants had been living in the same geographic area for at least 2 years before the start of the survey. Serum and urine samples were obtained from each participant at the beginning and at the end of the 6 months of the training program. The values of each mineral were determined by inductively coupled plasma mass spectrometry (ICP-MS). Additionally, the daily intake of each mineral was evaluated at both moments in time.

**Results:**

The daily concentrations of trace elements in the diet were similar at the start and the end of the training period without differences between groups. In serum, significant differences between groups were observed in As, Cd and Pb (*p* < 0.05). Attending to time effects, a significant difference was obtained in Pb (*p* < 0.05). In urine, significant differences between groups were obtained in all minerals (*p* < 0.05). According to training period, significant differences were observed in As, Be and Pb (*p* < 0.05). Finally, the group x time interaction revealed significant differences in As and Be (*p* < 0.05).

**Conclusions:**

Aerobic training may constitute a possibly effective method for increasing the elimination of Cd and Pb potentially toxic minerals from the body, especially among highly trained individuals.

## Introduction

Currently, environmental pollution produced by industries is one of society’s main concerns. Factories pour a large amount of possibly toxic metals for the organism, such as Arsenic (As), Berilium (Be), Cadmiun (Cd) or Lead (Pb), into the atmosphere [1]. These potentially toxic metals, in the air, water or food, can produce serious health problems.

Several health problems have been detected because of an abnormal level of As in the water, and different symptoms of gastrointestinal, dermatological, muscular, cardiovascular and neurological diseases have been observed [2]. Be is a potentially toxic mineral present in all industries around the world. The industrial spill of Be can cause, through inhalation, several pulmonary disorders, included cancer [3]. Cd is another common toxic mineral [4], showing severe toxicity in humans, often coming from smoking [5, 6]. The Cd concentration in the organism can provoke kidney damage, delay in growth, reproductive disorders, hypertension, and even cancer [7, 8]. Pb is used as an industrial element for many processes [9], and this can produce contamination in humans. But the experts cannot agree on what blood lead values are safe or unsafe. The effects of Pb on the cardiovascular, reproductive [10], renal [11] an immune [12] systems are well known; furthermore, bones and teeth can be affected by this element [13], which has been also identified as potentially carcinogenic [14]. Finally, a high Pb exposition during life has been associated with Alzheimer’s disease [15].

Preventive measures aimed at avoiding or minimizing the toxicity of these elements should become more and more important. In this respect, physical activity and training could be an interesting measure for preventing this toxicity. Previous studies have reported lower concentrations of Cd and Pb in high level Spanish athletes than recreational ones [16]. Later, Llerena et al. (2012) found a higher urinary excretion of Cd and another toxic elements in athletes with a high level of training than sedentary people [17]. Recently, Maynar-Mariño et al. (2018) have observed that serum concentrations of potentially toxic metals could be different, depending on the type of physical activity [18].. These results have been confirmed by Maynar et al. (2018). The authors have reported changes in potentially toxic trace element values in some biologic fluids, after an incremental test until exhaustion (serum and urine) [19].

However, no studies have been found about the effects of a six-month training program on potentially toxic metal concentrations. In all cases, current information about the long-term effect of continuous physical training on the serum or urinary concentrations of essential trace elements is limited and more research is required in this field.

Therefore, the aim of the present study was to observe if athletes (AG) present different concentrations of toxic trace elements (As, Be, Cd and Pb) with respect to sedentary people (CG) and if there are exercise-induced modifications in the serum and urinary concentrations as a result of a period of 6 months of intense, predominantly aerobic, physical training.

## Materials and methods

Materials and methods are the same as the ones described previously in Maynar et al., (2019) [20]. However, they will be presented again below.

### Participants

Fifty men participated in the present study, all living in the same city, Cáceres, Spain. Of these, twenty-four were Spanish national medium-distance runners (21 ± 4 years), recruited at the start of their training period, who formed the athletes’ group (AG). All of them had been competing in 1500 and 5000 m race modalities. They had been performing aerobic physical training regularly for the previous 2 years, developing an average volume of 100–120 km per week of rigorous training aimed at high-level competition. Their weekly training routines consisted of 3–4 days of aerobic continuous running and 2–3 days of aerobic-anaerobic fartlek or intense series.

The control group (CG) consisted of twenty-six untrained, non-sportsmen (21 ± 3 years), who only had been leading a normal, active lifestyle. Their physical activities consisted of recreational football, handball or basketball, recording a weekly volume of less than 2 h. The anthropometric characteristics of both groups are described in Table 1.

**Table 1:** Nutritional composition of the diets of both groups at the beginning and at the end of the survey

		Time		ANOVA (p)		
Nutritional intake		Baseline	6 months	Group effect	Time effect	***Group x Time interaction***
**Water** (g)	CG (*n* = 26)	1150.15 ± 400.22	1175.52 ± 418.15	0.21	0.15	0.43
	AG (*n* = 24)	1044.49 ± 288.31	1037.15 ± 334.41			
**Energy** (Kcal)	CG (*n* = 26)	2682.00 ± 550.1	2696.7 ± 600	0.02	0.14	0.30
	AG (*n* = 24)	2650.3 ± 414	2411.4 ± 325			
**Proteins** (g)	CG (*n* = 26)	115.5 ± 25.3	116.5 ± 25.5	0.53	0.45	0.88
	AG (*n* = 24)	118.0 ± 22.1	101.3 ± 22.5			
**Lipids** (g)	CG (*n* = 26)	100.7 ± 31.4	102.3 ± 29.50	0.22	0.22	0.30
	AG (*n* = 24)	97.1 ± 28.34	87.58 ± 25.71			
**Carbohydrates** (g)	CG (*n* = 26)	328.5 ± 55.33	327.50 ± 61.1	0.34	0.24	0.45
	AG (*n* = 24)	326.1 ± 52.39	304.5 ± 56.7			
**As** (12–300 mg/d)	CG (*n* = 26)	15.98 ± 14.13	16.26 ± 15.16	0.37	0.15	0.55
	AG (*n* = 24)	15.91 ± 15.61	15.48 ± 14.19			
**Be** (< 50 μg/d)	CG (*n* = 26)	8.24 ± 9.63	9.71 ± 10.70	0.18	0.25	0.62
	AG (*n* = 24)	8.40 ± 10.98	11.58 ± 9.28			
**Cd** (< 70 μg/d)	CG (*n* = 26)	23.05 ± 14.22	23.31 ± 16.97	0.25	0.57	0.87
	AG (*n* = 24)	26.02 ± 14.96	24.77 ± 15.00			
**Pb** (< 400 μg/d)	CG (*n* = 26)	190.65 ± 147.54	198.89 ± 151.48	0.68	0.55	0.78
	AG (*n* = 24)	188.62 ± 137.62	191.85 ± 127.20			

*CG* Control group, *AG* Athletes’ group. Values are presented as mean ± standard deviation

During the 6 months of the training period, the athletes ran a total of approximately 3500 km in training and competitions, varying the intensities from moderate (aerobic threshold) to high (anaerobic threshold or higher). The training was configured with 3–4 days of continuous running or fartlek and 2–3 days of more intense series, depending on whether there was a competition over the weekend. Low intensity, regenerative exercise was performed the day after a competition. The CG continued with their normal daily activities during the whole experimental period. None of the controls followed any specific physical training program.

A GPS pack equipped with pulsometers (Polar M430. Norway) was used to track the training loads during the survey. The GPS were lent to the sportsmen at the beginning of the survey and the researchers recorded and analyzed their training routines every week.

All the participants had been living in the same city (Cáceres, Spain) for at least 2 years. The present study was approved by the bioethics committee of the University of Extremadura according to the Helsinki Declaration ethic guidelines of 1975, updated at the World Medical Association Assembly in Fortaleza 2013, for investigations involving human subjects. All the participants were explained the purpose of the study and written their informed consent.

### Anthropometric measurement

The morphological characteristics of the participants were measured in the morning and always at the same time and under identical conditions. Body height was measured to the nearest 0.1 cm using a wall-mounted stadiometer (Seca 220. Hamburg. Germany). Body weight was measured to the nearest 0.01 kg using calibrated electronic digital scales (Seca 769. Hamburg. Germany) in nude, barefoot conditions. Sum of 6 skinfolds (∑6) (abdominal, suprailiac, tricipital, subscapular, thigh and calf skinfolds) were measured. Skinfold thicknesses were measured with a Harpenden caliper (Holtain Skinfold Caliper. Crosswell, UK). All measurements were made by the same operator, skilled in kinanthropometric techniques, in accordance with the International Society for the Advancement of Kinanthropometry recommendations [21]. Heart rate and blood pressure were determined using an automatic sphygmomanometer (Omron HEM-780. Osaka. Japan) by a skilled technician, always after a five-minute rest period in a supine position.

### Nutritional evaluation

All participants completed a dietary questionnaire in order to ensure that they were not taking any vitamins, minerals or other supplements and in order to guarantee that they were following a similar diet. The questionnaire consisted of a 3-day, daily nutritional record, filled out on two pre-assigned weekdays and on one weekend day.

On each day, all participants recorded the amount (in grams) of each food consumed in every meal ingested on every one of the 3 days. Once completed, every questionnaire compiled the total amount of each food consumed, grouped by meals. Then the nutritional composition of their diets was evaluated using different food composition tables [22–24]. These tables contain nutritional information about all kinds of foods. The nutritional questionnaires were applied at the start and at the end of the study period. None of the participants followed a specific diet, nutritional plan or specific supplementation during the whole survey. All of them were allowed to hydrate freely during the study.

### Maximal exercise test until exhaustion

An exercise test was used to evaluate the performance variables for each participant. The test consisted of running on a treadmill (Powerjoc. UK) until voluntary exhaustion. The ergospirometric and cardiovascular variables were measured using a gas analyzer (Metamax. Cortex Biophysik. Gmbh. Germany) and a Polar pulsometer (Polar M430. Norway). To guarantee a warm-up phase before the test, all participants ran progressively for 15 min, ending at the initial speed of the test. Then, the participants performed the exercise test. The CG participants performed 5 min at 6 km/h, 5 min at 7 km/h and 5 min at 8 km/h to ensure a proper warm-up phase. Athletes ran at 8, 9 and 10 km/h respectively. The participants then performed the exercise test. The protocol consisted in running incrementally in stages, until voluntary exhaustion (no possibility of continuing to run) starting at an initial speed of 8 km/h for controls and 10 km/h for athletes and increasing the speed by 1 km/h every 400 m, with a stable slope of 1%. The anaerobic threshold was determined using the triphasic model described by Skinner and McLellan using the ventilatory parameters [25]. This test was used to run a sufficient distance in order to achieve the same physiological changes which should be expected to occur in a field test. All tests were performed in the morning (between 10 and 12 a.m.). Training intensity and volume were reduced the two previous days applying a regenerative load in order to avoid fatigue in the physical tests. The exercise tests were performed at the beginning and at the end of the experimental period, with the time and conditions being the same for each participant.

### Sample collection

Samples were collected after a fasting period of 8 h. At nine o’clock in the morning 5 mL of venous blood were drawn from each participant using a plastic syringe fitted with a stainless-steel needle. The blood samples were collected in a metal-free polypropylene tube (previously washed with diluted nitric acid). Then, the blood samples were centrifuged at 3000 rpm for 15 min at room temperature to separate the serum. Once isolated, the serum was aliquoted into an Eppendorf tube (previously washed with diluted nitric acid) and was conserved at − 80 °C until further analysis. Morning midstream urine samples were obtained from all subjects and were collected in polyethylene tubes previously washed with diluted nitric acid and frozen at − 80 °C until analysis. Prior to analysis, the samples were thawed and homogenized by shaking. This protocol was applied at the beginning and at the end of the experimental period.

### Experimental design

#### Urinary creatinine determination

Creatinine concentrations were measured, in duplicate, in all urine samples to determine different dilution degrees [26], using Sigma’s Creatinine 555–A kit and a UNICAM 5625 spectrophotometer.

### Serum and urinary trace element determination

#### Sample preparation

As, Be, Cd and Pb analyses were performed by inductively coupled plasma mass spectrometry (ICP-MS). To prepare the analysis, the organic matrix was decomposed by heating it for 10 h at 90 °C after the addition of 0.8 mL HNO_3_ and 0.4 mL H_2_O_2_ to 2 mL of serum or urine samples. The samples were then dried at 200 °C on a hot plate. Sample reconstitution was carried out by adding 0.5 mL of nitric acid, 10 μL of Indium (In) (10 mg/L) as the internal standard, and ultrapure water to complete 10 mL.

#### Standard and reference material preparation

Reagent blanks, element standards and certified reference material (Seronorm, lot 0511545, AS Billingstand, Norway) were prepared identically, and used for accuracy testing. Before the analysis, the commercial control materials were diluted according to the recommendation of the manufacturer.

#### Sample analysis

Digested solutions were assayed with an ICP-MS Nexion model 300D (PerkinElmer, Inc., Shelton, CT, USA) equipped with a triple quadrupole mass detector and a reaction cell/collision device that allows operation in three modes: without reaction gas (STD); by kinetic energy discrimination (KED) with helium as the collision gas; and in reaction mode (DRC) with ammonia as the reaction gas. Both collision and reaction gases such as plasmatic argon had a purity of 99.999% and were supplied by Praxair (Madrid, Spain). Two mass flow controllers regulated gas flows. The frequency of the generator was free-swinging and worked at 40 Mhz. Three replicates were analyzed per sample. The sample quantifications were performed with indium (In) as the internal standard. The values of the standard materials of each element (10 μg/L) used for quality controls were in agreement with intra and inter-assay variation coefficients of less than 5%.

### Statistical evaluations

Statistical analyses were carried out with IBM SPSS Statistics 22.0 for Windows. The results are expressed as means ± standard deviations. Normality was tested with the Shapiro-Wilk test. A two-way ANOVA was used to show differences between study variables. The level of significance was set at *p* < 0.05.

## Results

### Nutritional evaluations

Table 1 presents the results of the nutritional analysis of the participants’ diets.

None of the participants followed any special diet like, for example, vegetarian or vegan. The only significant difference was observed between groups in kilocalorie intake (*p* < 0.05). There were no significant differences in mineral intake.

### Anthropometric and ergospirometric characteristics of participants

Table 2 shows the anthropometric and ergospirometric data on athletes and controls.

**Table 2:** Anthropometric and ergospirometric results of controls and athletes at the start and end of the study period

		Time		ANOVA (p)		
Parameters		Baseline	6 months	Group effect	Time effect	***Group x Time interaction***
**Total Weight** (Kg)	CG (*n* = 26)	76.94 ± 11.07	77.62 ± 12.14	0.001	0.2	0.11
	AG (*n* = 24)	65.55 ± 7.55	64.73 ± 7.83			
**∑6 Skinfold** (mm)	CG (*n* = 26)	62.76 ± 12.23	63.02 ± 13.05	0.001	0.08	0.13
	AG (*n* = 24)	48.74 ± 11.17	46.59 ± 8.89			
**Rest HR** (b/min)	CG (*n* = 26)	65.12 ± 12.42	67.34 ± 11.71	0.001	0.13	0.21
	AG (*n* = 24)	52.12 ± 12.42	49.87 ± 9.27			
**HR max** (b/min)	CG (*n* = 26)	196.33 ± 7.55	197.41 ± 8.01	0.42	0.51	0.72
	AG (*n* = 24)	193.69 ± 7.85	194.30 ± 7.50			
**VO**_**2**_ **max** (ml/kg/min)	CG (*n* = 26)	45.72 ± 7.51	46.32 ± 8.16	0.001	0.2	0.31
	AG (*n* = 24)	66.46 ± 10.12	67.94 ± 8.10			
**VE max** (L/min)	CG (*n* = 26)	98.66 ± 11.47	99.26 ± 16.63	0.001	0.42	0.25
	AG (*n* = 24)	137.95 ± 55.34	129.00 ± 26.92			

*CG* Control group, *AG* Athletes’ group, *HR* Heart rate. *VO*_*2*_ Oxygen uptake, *VE* Pulmonary ventilation

Values are presented as mean ± standard deviation

When examining the group effect, significant differences were observed in total weight, ∑6 skinfolds, resting HR, VO_2_ max and VE max (*p* < 0.01). Attending to time effect and group x time interaction, no significant differences were observed.

### Serum concentrations of metals

**Fig. 1:**
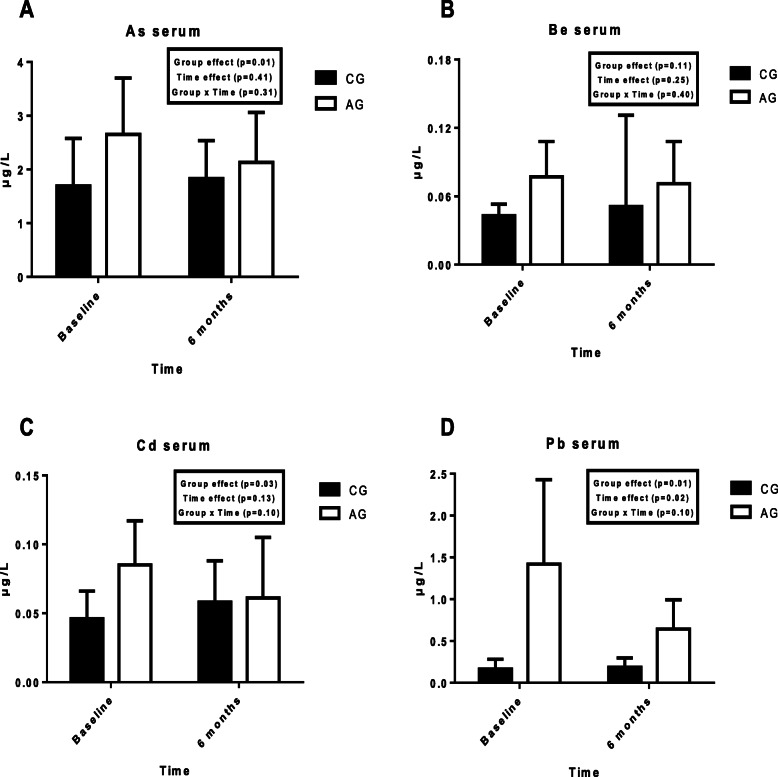
Serum concentrations of toxic mineral in controls and athletes at the start and the end of the 6 months of the study; **a** = As; **b =** Be = **c** = Cd; **d** = Pb; As = arsenic; Be = beryllium; Cd = cadmium; Pb = lead; AG: athletes group; CG: control group

Figure 1 shows the serum concentrations of each metal at the start and at the end of the training period in both groups.

Significant differences were observed between groups, with higher values of As, Cd, Pb in AG (*p* < 0.05). Attending to time effect, a significant difference in Pb was observed (*p* < 0.05). No significant differences were observed in the group x time interaction.

### Urinary concentrations of metals

**Fig. 2:**
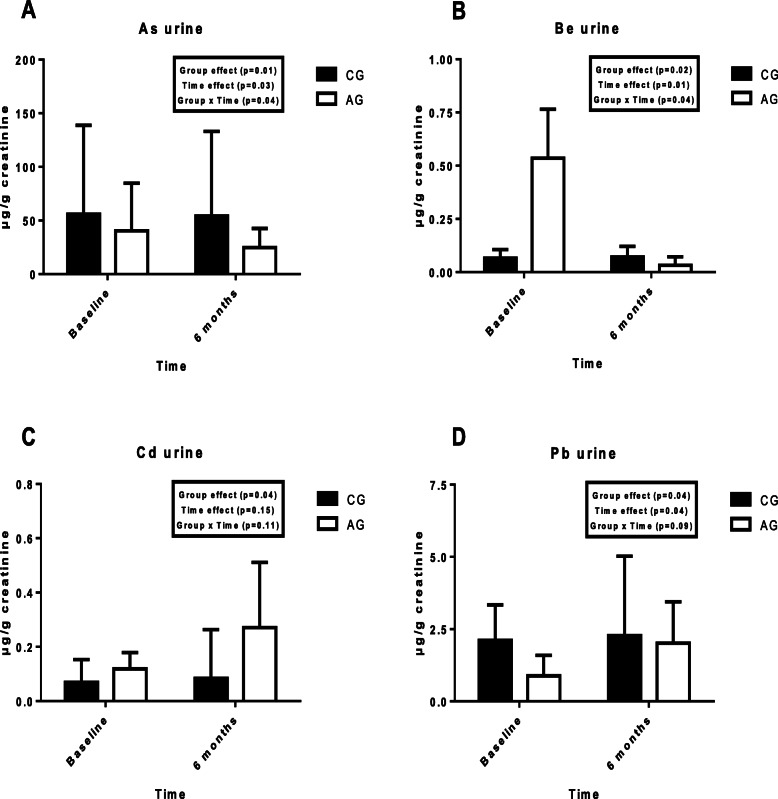
Urinary concentrations of toxic minerals in controls and athletes at the start and the end of the 6 months of the study; **a** = As; **b =** Be = **c** = Cd; **d** = Pb; As = arsenic; Be = beryllium; Cd = cadmium; Pb = lead; AG: athletes group; CG: control group

Figure 2 shows the urinary concentrations of each metal in both groups at the start and at the end of the training period. The results are expressed with creatinine corrections (in μg/g creatinine).

When examining the group effect, significant differences were observed in the urinary excretion of all minerals (*p* < 0.05). According to the training period, significant differences were obtained in As, Be and Pb (*p* < 0.05). Finally, in the group x time interaction significant differences were observed in As and Be (*p* < 0.05).

## Discussion

The aim of the present study was to observe the possible differences in toxic metal concentrations between athletes and sedentary people, in serum and urine. Also, we tried to determine the effects of 6 months of physical training, predominantly aerobic, on these values.

Typically, middle and long distance runners perform a high volume of kilometers to improve their endurance capacity, and to get excellent results in competition [27]. This author affirms that the clear relationship between training load and performance encourages athletes to attempt progressively heavier training loads in the quest for the small (< 2%) improvements in performance that produce competitive results at the highest levels. This training load produces significant changes in metabolism, and a diminution in total weight and fat percentage.

Moreover, nutritional intake is another important parameter to control, because food is one of the sources of exposure to these toxic elements [22]. Our results showed a similar estimated intake in all toxic minerals analyzed (Table 1). However, the AG had a higher total energy intake than CG (*p* < 0.05).

First, all the serum and urinary concentrations of toxic metals obtained in this study were in the normal range, so that none of the subjects presented a health risk [28, 29]. Creatinine production is proportional to the muscle mass of the individual and in the absence of any renal pathology, creatinine excretion rates are constant, and not modified by physical exercise or by variations in catabolism. This is essential because overestimations of mineral values may occur if the urine is at a high concentration [30].

All participants lived in the same region and were the same age, this helped to avoid several factors which could have influenced the results. The results showed significant differences between groups in serum values of As, Cd and Pb, with a higher concentration in AG (*p* < 0.05). The high level of training in athletes could provoke an excess by inhaling more air, drinking more water, or eating more food. In this way, an increase could be possible in toxic metals ingested [18]. Previous studies have reported a high probability of sportsmen presenting higher serum concentrations of Pb than sedentary individuals, because of pollution [31]. In relation to time effect, the ANOVA showed significant differences in Pb (*p* < 0.05), similar to those previously obtained [32].

Bartolomé et al. (2016) obtained similar results in the urinary excretion of As after a paddle match [33]. Likewise, Maynar et al. (2018) reported a rise in serum and a decrease in urine concentrations of this element after an incremental exercise test [19]. A recent study reported higher As erythrocyte levels in highly trained athletes [34]. Thus, health problems are linked to abnormally high As levels in water [2]. The results of this research suggest that highly trained athletes living in areas with a high concentration of As in the water could be at risk of health disorders that could affect their sport performance.

On the other hand, no changes in serum Be were found, but a drop was observed in the excretion in urine. Be acts as an inhibitor of various enzymes related to physical activity, such as glycogen synthase kinase-3 (GSK-3) or cyclin-dependent protein kinase (cdc2) [35]. This could decrease post-training Mg bioavailability [36]. Besides, the mentioned enzymes could bind Be instead of Mg. This phenomenon could be the reason for Be urine concentrations falling after training. Parallel to these findings, recent research found a decrease in urine Be levels after an exercise test until exhaustion in athletes (Maynar et al., 2018). Thus, a training induced adaptation process in the organism could elicit a decrease in the urinary excretion of Be.

Regarding the Cd results, higher serum concentrations and urinary excretion were found in AG than CG. The main effect was observed between groups. Grijota et al. (2019) reported lower Cd concentrations in sportsmen’s erythrocytes [34]. In addition, the decreased serum Cd obtained in this survey among AG participants after the training period is similar to that observed by Kara (2012). This author noted a significant decrease in serum Cd after 3 months of football training in young boys [37]. It has been reported that physical exercise produces a trace element redistribution among different tissues in the organism, which could explain the decreased serum values [38]. In the same way, it has been reported that Cd could be excreted in sweat, even more than in urine [39, 40]. This excretion by sweating could be a preventive response of the organism against Cd toxicity.

Furthermore, previous studies found a significant increase in Cd excretion after an exercise until exhaustion [19], and a higher urinary concentration in athletes with respect to the control group [17]. In this respect, major excretion could be expected after 6 months of physical training, but no time effect was observed. In addition, the relevant role of the kidney in the organic regulation of toxic elements could cause an adaptive process to perform these functions [41]. Large concentrations of this element could cause hepatic and renal damage [42], in the worst cases, some months, or even years, being necessary to achieve its excretion, producing some health disorders [43]. Finally, the increased excretion of Cd after 6 months of physical training, could be the organism’s response to improve the elimination of this toxic metal, avoiding its accumulation. Although more research is needed, these results could consider exercise as a preventive therapy in areas with a high level of pollution.

In relation to Pb, the serum concentration of this element decreased at the end of the training period, and an increase in the urinary excretion was observed. Previous studies in subjects exposed to Pb, reported decreases in blood Pb after aerobic training [32]. In urine, similar results were obtained previously [17]. This could indicate, as in the case of Cd, a natural strategy (physical activity) to reduce the Pb levels in the body, especially in industrialized areas or big cities, due to the high amounts of this mineral in the air.

This idea is supported by data obtained by Rodriguez Tuya et al. (1996), who reported significantly lower concentrations of Pb in athletes living in Madrid, compared to moderately trained subjects [16]. These athletes had been training in this city for several years, following a high-volume competitive training routine.

Correlations between blood and sweat Pb levels have been previously reported [44]. After strenuous exercise, higher Pb values have been observed in sweat than in urine [45]. In this respect, another investigation showed a significant increase in the excretion of Pb by sweating after acute exercise to exhaustion [46]. Thus, as in the case of Cd, this excretion by sweating could be a preventive response of the organism against Pb toxicity [19].

Some limitations of the study should be noted. First, we did not take into account the possible differences between organic and non-organic food intake, and filtration of water between groups. Second, we are not able to provide information about water analysis, because of the values of toxic metals were under the limit of detection. However, the exposures (air, water, food source, etc.) were similar during this observation period. Finally, futures research should explore the content in tissues, such as adipose, for a more stable assessment of levels in vivo.

## Conclusions

Six months of an aerobic training program produced an increase in the urinary excretion of Cd, suggesting a response of the organism to avoid the accumulation of this potentially toxic element in body cells.

Also, this program induced a decrease in serum values of Pb, accompanied by an increased excretion in urine of this element, in order to reduce its concentration in the body.

The results report that aerobic physical training can be an efficient method for removing some potentially toxic trace elements such as Cd and Pb, a fact that is very important for preventing the exposition caused in people living in industrial areas. More studies are needed in order to investigate the potential benefits of physical exercise on the excretion of potentially toxic metals by the organism, including loss from sweating.

## Data Availability

All data generated or analyzed during this study are included in this published article.

## Abbreviations

As: Arsenic
AG: Athletes group
Be: Beryllium
CG: Control Group
Cd: Cadmium
Pb: Lead
Mg: Magnesium
Km: Kilometer
Mg: Milligrams
mL: Milliliter
L: Liter
ICP-MS: Inductively Coupled Plasma Mass Spectrometry
GPS: Global Positioning System
Rpm: Revolutions per minute
HNO_3_: Nitric Acid
H_2_O_2_: Hydrogen Peroxide
Σ6: Sum of 6 skinfolds
HR: Heart Rate
VO_2_: Oxygen Consumption
VE: Pulmonary Ventilation
