# Correction to: Serum and urinary concentrations of arsenic, beryllium, cadmium and lead after an aerobic training period of six months in aerobic athletes and sedentary people

**DOI:** 10.1186/s12970-021-00429-1

**Published:** 2021-04-14

**Authors:** Diego Muñoz, Francisco J. Grijota, Ignacio Bartolomé, Jesús Siquier-Coll, Víctor Toro-Román, Marcos Maynar

**Affiliations:** 1 Sport Sciences Faculty, University of Extremadura, Avenida de la Universidad s/n, 10003 Cáceres, Spain; 2 Faculty of Language and Education, Antonio de Nebrija University, Madrid, Spain

**Correction to: J Int Soc Sports Nutr 17, 43 (2020)**

**https://doi.org/10.1186/s12970-020-00372-7**

The original article [1] contains an incorrect affiliation for co-author, Francisco J. Grijota.

The correct affiliation can be viewed in this correction article (Faculty of Language and Education, Antonio de Nebrija University, Madrid, Spain).
